# Computational prediction of new therapeutic effects of probiotics

**DOI:** 10.1038/s41598-024-62796-4

**Published:** 2024-05-24

**Authors:** Sadegh Sulaimany, Kajal Farahmandi, Aso Mafakheri

**Affiliations:** 1 Social and Biological Network Analysis Laboratory (SBNA), Department of Computer Engineering, University of Kurdistan, Sanandaj, Iran; 2 Department of Industrial and Environmental Biotechnology, National Institute of Genetic Engineering and Biotechnology (NIGEB), Tehran, Iran

**Keywords:** Probiotics, Disease, Link prediction, *Lactobacillus jensenii*, Probio database, Computational models, Nutrition therapy

## Abstract

Probiotics are living microorganisms that provide health benefits to their hosts, potentially aiding in the treatment or prevention of various diseases, including diarrhea, irritable bowel syndrome, ulcerative colitis, and Crohn’s disease. Motivated by successful applications of link prediction in medical and biological networks, we applied link prediction to the probiotic-disease network to identify unreported relations. Using data from the Probio database and International Classification of Diseases-10th Revision (ICD-10) resources, we constructed a bipartite graph focused on the relationship between probiotics and diseases. We applied customized link prediction algorithms for this bipartite network, including common neighbors, Jaccard coefficient, and Adamic/Adar ranking formulas. We evaluated the results using Area under the Curve (AUC) and precision metrics. Our analysis revealed that common neighbors outperformed the other methods, with an AUC of 0.96 and precision of 0.6, indicating that basic formulas can predict at least six out of ten probable relations correctly. To support our findings, we conducted an exact search of the top 20 predictions and found six confirming papers on Google Scholar and Science Direct. Evidence suggests that *Lactobacillus jensenii* may provide prophylactic and therapeutic benefits for gastrointestinal diseases and that *Lactobacillus acidophilus* may have potential activity against urologic and female genital illnesses. Further investigation of other predictions through additional preclinical and clinical studies is recommended. Future research may focus on deploying more powerful link prediction algorithms to achieve better and more accurate results.

## Introduction

The use of probiotics that were initially restricted for antibiotic-associated diarrhea now extends as powerful anti-allergic and anti-inflammatory factors^1^. For example, recent studies have shown that probiotics may help improve immune function, protect against hostile bacteria to prevent infection and improve digestion and absorption of food and nutrients. They can act as gut-beneficial bacteria that create a physical barrier against unfriendly bacteria. Therefore, the potential benefits of probiotics have been seen in the treatment or prevention of many conditions, such as diarrhea, irritable bowel syndrome, ulcerative colitis, and Crohn’s disease^2^.

Probiotics are living microorganisms that offer various health benefits to their hosts^3^. Their antibacterial effect is attributed to producing organic acids, ethanol, hydrogen peroxide, or bacteriocins^4,5^. According to WHO, once antibiotics no longer work due to antibiotic resistance, probiotics are considered the next most significant immune defense system^6^. In analyzing nearly 300 meta-analyses published from 2000 to 2020 about the effects of probiotics in the prevention and treatment of diseases, probiotics were 79% effective in the prevention or treatment of various diseases. Only 21% of these papers reported ineffectiveness of probiotic strains, and no studies have demonstrated the detrimental effects of probiotics^7^.

Link prediction is a widely researched area in biological and medical science, aimed at discovering potential connections between related entities such as drug-target, protein–protein, gene-disease interactions, etc.^8^. This approach can save researchers time and resources by identifying promising biological links and providing new insights for further investigation. However, there is a lack of research on link prediction in computational biology to identify new probiotic-disease relationships. While a few studies have investigated the prediction of specific probiotic effects^9^, there is currently no general research available that proposes new links between current probiotics and diseases. Therefore, conducting a new study to identify unregistered probiotic effects on diseases could be a worthwhile endeavor.

Our study aims to contribute to the field by creating and visualizing a bipartite network of probiotics and their associated diseases. We then employ computational techniques to predict the most probable probiotic-disease pairs and evaluate our results through both computational analysis and a search of recent literature. To achieve this, our paper is structured as follows: Firstly, we review the existing literature on the associations between probiotics and diseases. We then provide a detailed explanation of the computational background of bipartite link prediction. Following that, we describe our data-gathering procedure and the computational method used to perform balanced link prediction in bipartite graphs. The result and discussion section will present the outcomes of our research and provide justification and reasoning for our findings. Finally, the conclusion section will summarize our paper and outline future research directions and extensions.

## Literature review

To propose a computational method for predicting new relations between probiotics and diseases, it is important to provide a concise overview of the effects of probiotics on diseases. Afterward, the concept of link prediction can be introduced, focusing on its application to bipartite graphs, which are well-suited for modeling probiotic-disease relations.

### Probiotics’ effects on diseases

Numerous studies have been conducted to evaluate the effects of probiotic supplements on a wide range of diseases, many of which have demonstrated their effectiveness. Table 1 summarizes the efficacy of probiotics on some of the most common diseases in important categories.

**Table 1:** Important categories of diseases in which probiotics may be effective on.

Disease categories	Some common diseases	The effectiveness of probiotics	Some effective probiotic strains
Gastrointestinal diseases	Diarrhea	Having immunostimulatory activity and restoring gut microflora to the balance situation^53,54^	*Limosilactobacillus reuteri* ATCC 55,730^53,55^ and *Saccharomyces boulardii* ^56,57^
	Irritable bowel syndrome (IBS)	Reducing intestinal cytokine secretion and improving epithelial barrier function^58^	*Lactobacillus rhamnosus* ^59^ *Escherichia coli* Nissle 1917^60^
	Inflammatory Bowel Disease (IBD)	Lowering the NF-κB DNA binding activity, decreasing the accumulation of leukocytes, and reducing the production of IL-6 and TNF-α	*Faecalibacterium prausnitzii* ^61^ Lactobacillus rhamnosus CNCM I-3690^62^
Respiratory diseases	Influenza	Decreasing viral loading in the lung, and altering the immune responses	*Bacillus subtilis* 3^63^, *Lactobacillus rhamnosus* M21^63^, *Bacillus subtilis* PY79^64^
	Covid-19	Restoring gut microbiota balance, decreasing the risk of secondary infection due to bacterial translocation^65^, antiviral properties^36^, enhancing the protective functions of the intestinal and lung barriers and maintaining balance, through the promotion of regulatory T cells, bolstering antiviral defenses, and decreasing the levels of pro-inflammatory cytokines^37^	*Lactobacillus rhamnosus* GG^37^
	Pneumonia	limiting the colonization of pathogen species or improving host immune defenses^38^	*Lactobacillus acidophilus* LA-5, *Lactobacillus plantarum*, *Bifidobacterium lactis* BB-12, and *Saccharomyces boulardii* ^66^
Metabolic diseases	Diabetes	Lowering inflammation and oxidative stress within the cells of the pancreas^42,67^, and preventing the destruction of β-pancreatic cells^42,43^	*Bifidobacterium adolescentis* ^68^
	Obesity	Increasing anorexigenic neuropeptide expression^69^, and hypothalamic control of appetite^70^	*Lactobacillus plantarum*, *Lactobacillus gasseri* ^49^, *Methanobrevibacter smithii* and *Blautia hydrogenotrophica* ^71^
Mental illnesses	Depression and anxiety	Controlling important neurotransmitters and regulating inflammatory markers^72^	A combination of *Lactobacillus reuteri* NK33 and *Bifidobacterium adolescentis* NK98^52^ a combination of *Lactobacillus helveticus* R0052 and *Bifidobacterium longum* R0175^52^

Probiotics are effective in treating gastrointestinal disorders by improving intestinal mucosal barrier function, increasing mucus production, reducing inflammation, and restoring normal bowel movements^10,11^. Probiotics can also prevent or treat most types of diarrheas. However, their efficacy depends on several factors, including the strain type, antimicrobial and anti-inflammatory properties of the probiotic, and dosage^12–14^.

Irritable bowel syndrome (IBS) is another common disease in this category, which is an unexplained brain-gut disorder with multiple contributing factors. Numerous studies have demonstrated that gut microbiota may be crucial in improving subjective symptoms in IBS patients^15^.

A meta-analysis of 15 human studies, involving 1793 patients with IBS, found that probiotic therapy reduced pain and symptom severity scores^16^. Inflammatory Bowel Disease (IBD) is a multifactorial disease, like IBS, that encompasses Crohn’s disease (CD), ulcerative colitis (UC), and nonspecific colitis^17^. The manifestation and progression of IBD are influenced by several factors, including genetics, environmental agents, disruption in immunological responses, permeability of the gut barrier, and microbiota composition^18^. Research has shown that products derived from *Faecalibacterium prausnitzii* improved the tightness of the gut barrier in mice suffering from colitis, according to Carlsson et al.^19^. In addition, other studies on animal models have demonstrated that *Bifidobacterium infantis* and *Bifidobacterium bifidum*, through their immunomodulatory activities, can decrease inflammation and clinical symptoms of colitis^20,21^.

Besides, probiotics play a significant role in preventing and treating respiratory diseases^22,23^ and may serve as a practical alternative for promoting recovery^24,25^. Research suggests that probiotics can stimulate respiratory immunity by enhancing T regulatory function in the airway and improving resistance to bacterial and viral respiratory tract infections^26–28^. Notably, *Bifidobacterium* and *Lactobacillus* have been identified as effective treatments for rotavirus infection in both animals and humans^29–31^.

Recent research has demonstrated that probiotics can treat allergic rhinitis (AR), a chronic respiratory disease. *Lactobacillus paracasei* LP-33 is one probiotic strain found to alleviate at least one symptom in patients with AR. Probiotics have also effectively treated other respiratory diseases, including influenza, COVID-19, and pneumonia. For example, *Bacillus subtilis* 3 produces an antibiotic called aminocoumarin that can eliminate many pathogens while also enhancing host resistance^32^. In addition to its effectiveness in preventing and treating influenza, this strain has also been reported to help prevent and treat bacterial infections in both animal and human models^33–35^. Given the antiviral effects of probiotics against other viruses, they could serve as a complementary treatment against SARS-CoV-2^36^. Human studies have demonstrated that probiotics, particularly *Lactobacillus rhamnosus* GG, can improve intestinal and lung barriers, maintain homeostasis, enhance antiviral defense, and reduce pro-inflammatory cytokines in respiratory and systemic infections by increasing regulatory T cells. These immunomodulatory agents may be useful for those at risk of contracting COVID-19 or who have already been infected^37^. Moreover, probiotic therapy is a promising non-antibiotic method for protecting the host microbiota balance and preventing Ventilator-associated pneumonia (VAP). By limiting the colonization of pathogenic species or enhancing host immunity, probiotics may help to reduce the incidence of VAP^38^.

Research has also demonstrated the positive effects of probiotics on metabolic diseases. Insulin resistance, diabetes onset, and diabetic retinopathy pathogenesis are all associated with oxidative stress^39–41^. Some probiotic strains are effective in reducing inflammation and oxidative stress in pancreatic cells and inhibiting the destruction of β-pancreatic cells^42–44^. Numerous meta-analyses have reported that probiotic supplements can lead to weight loss and improvements in body mass index (BMI)^45–48^. However, different species of *Lactobacillus* may have different effects on weight in both humans and animals^49^, leading to contradictory results on the efficacy of probiotics in weight alterations, which may be attributed to differences in probiotic strains and host factors.

Further, the gut-brain axis suggests that intestinal and mental health are interconnected, leading to the hypothesis that probiotic supplementation may benefit mental disorder management and regulation^50^. Studies have confirmed this notion. One trial with 86 students found that daily probiotic supplementation for 28 days improved panic anxiety, neurophysiological anxiety, worries, and mood regulation^51^. Another randomized clinical trial involving 156 healthy adults with subclinical symptoms of depression, anxiety, and insomnia demonstrated that probiotic supplements containing a mix of *Lactobacillus reuteri* NK33 and *Bifidobacterium adolescentis* NK98 for 8 weeks improved mental health and sleep^52^. Table 1 summarizes the findings on the effectiveness of probiotics for diseases.

Studying the impact of each probiotic strain on unexplored diseases requires extensive and expensive research that includes preclinical and clinical trials for evaluating safety and efficacy. Given the vast number of probiotic strains and diseases, this process can be time-consuming and labor-intensive. To facilitate the validation of potential links between probiotics and disease, more efficient computational methods are needed to reduce the costs and streamline comprehensive trials.

Previous research has demonstrated the successful application of computational models in various fields, such as drug-target interactions^73^, microbe-disease prediction^74^, and gene-disease association prediction^75–77^, as well as miRNA and disease prediction^78–80^, and protein structure prediction. However, to date, no study has focused on predicting the potential therapeutic effects of probiotic strains on various diseases. Therefore, our research aims to determine which probiotic strains have the greatest therapeutic potential for treating different diseases.

### Link prediction

**Figure 1:**
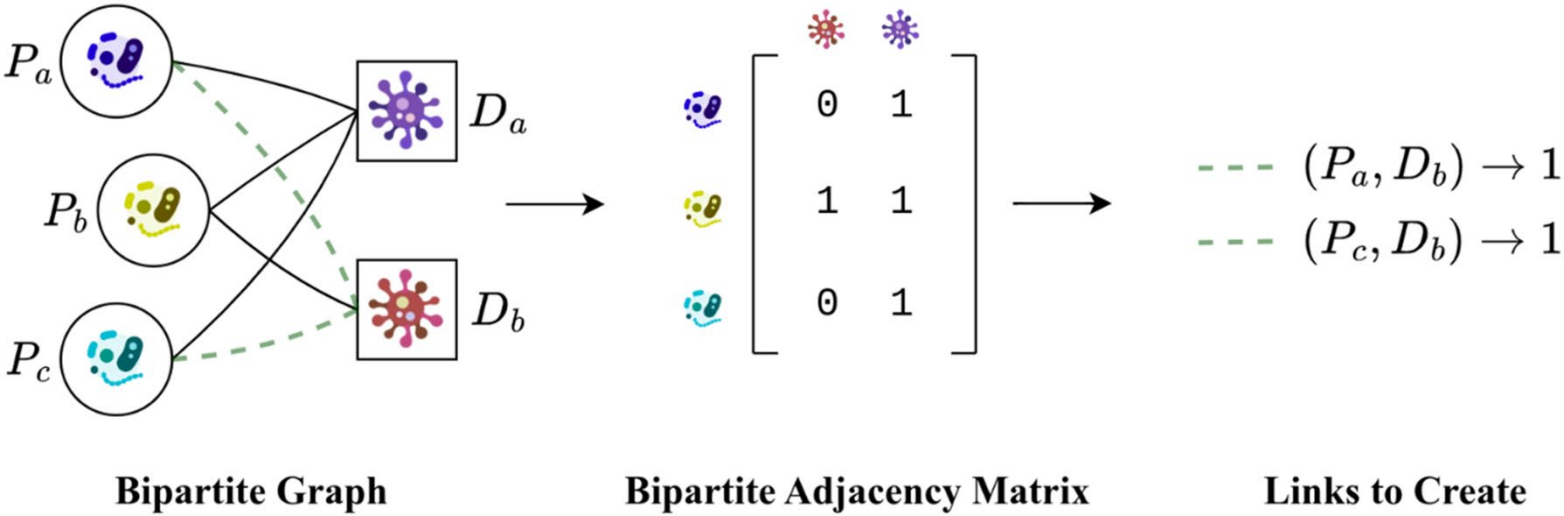
An illustration of basic applications of link prediction for adding probable relations in a network that has been modeled with an adjacency matrix.

Link prediction refers to the task of identifying the most likely future connections in a network. This problem can take different forms depending on the types of relationships and network configurations, and various link prediction methods exist^81^. In our study, we represent the network structure using an adjacency matrix where existing links are represented as 1. To predict future or missing links, we apply link prediction ranking algorithms to the zero entries of the matrix to determine the most probable connections, as shown in Fig. 1.

**Figure 2:**
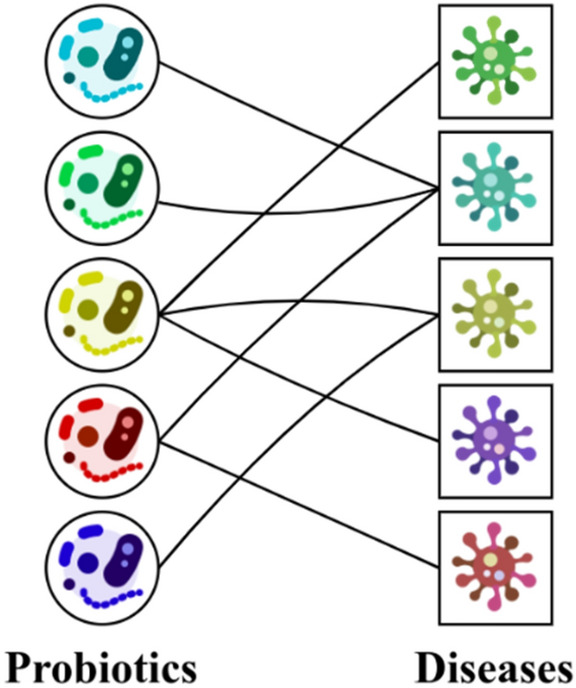
In a bipartite probiotic-disease network, we just consider predicting the relations between parts. This type of network has its modeling and formulas as well.

In this study, we employ link prediction to predict new associations between probiotics and diseases. To accomplish this, we utilize a specialized network model that only considers the relationships between two entities within a network, rather than within each entity. This approach allows us to concentrate specifically on the connections between probiotic-disease pairs, as opposed to probiotic-probiotic or disease-disease relationships, as shown in Fig. 2.

As the investigated network exhibits a bipartite structure, custom versions of link prediction formulas are required. Our approach employs basic algorithms, mainly based on considering the neighbors of a node in a network. Specifically, the methods employed in this study are Common Neighbors (CN), Jaccard coefficient (JC), Adamic/Adar (AA), and Preferential Attachment (PA)^82^. CN, JC, and AA use the neighborhood of two unconnected nodes to determine the likelihood of their connection. At the same time, PA considers broader characteristics of the nodes, their degree, to decide the importance of forming new links.

Further, Table 2 presents the main formulas used to predict new relations in a bipartite network in a concise and easy-to-understand manner. The formals are the improved version of^83^ that consider similar conditions for each part of the network, probiotic or disease. Order references such as Bakhtiari et al. (2020a) just consider the ranking from one side. For example, for the probiotics side, only consider the intersection of probiotics and the probiotics connected to the target disease. Nevertheless, we rank the relations from both sides: probiotics neighbors and diseases neighbors for the more accurate and stable computation.

**Table 2:** Special link prediction methods in the bipartite network of probiotic-disease for finding the most probable relations.

Method name	Abbreviation	Easy-to-understand description
Common Neighbor	CN	Prioritizes non-connected nodes with the maximum number of shared neighbors to increase their ranking
Jaccard Coefficient	JC	The normalized version of CN that takes into account the total number of neighbors for two potential nodes as well as the neighbors they have in common
Adamic Adar	AA	Similar to RA, this method pays more attention to common neighbors with lower degrees. However, using a Log scale facilitates easier handling and compression of very large or small values
Preferential Attachment	PA	Assigns higher rankings to two non-connected nodes with a greater number of neighbors, whether shared or not

Method	Ranking formula	
CN	$\left( {\left| {\Gamma \left( x \right) \cap \Gamma \left( {\Gamma \left( y \right)} \right)} \right| + \left| {\Gamma \left( y \right) \cap \Gamma \left( {\Gamma \left( x \right)} \right)} \right|} \right)$)/2	
JC	$\frac{{\left( {\left| {\Gamma \left( x \right) \cap \Gamma \left( {\Gamma \left( y \right)} \right)} \right| + \left| {\Gamma \left( y \right) \cap \Gamma \left( {\Gamma \left( x \right)} \right)} \right|} \right)/2}}{{\left| {\Gamma \left( x \right) \cup \Gamma \left( y \right)} \right|}}$	
AA	$\left( {\sum\limits_{{z \in \Gamma \left( y \right) \cap \Gamma \left( {\Gamma \left( x \right)} \right)}} {\frac{1}{{\log \left| {\Gamma \left( z \right)} \right|}}} + \sum\limits_{{z \in \Gamma \left( x \right) \cap \Gamma \left( {\Gamma \left( y \right)} \right)}} {\frac{1}{{\log \left| {\Gamma \left( z \right)} \right|}}} } \right)/2$	
PA	$\left| {\Gamma \left( x \right)} \right| \times \left| {\Gamma \left( y \right)} \right|$	

First part of the table introduces the names and abbreviations and a simple short description of the method. The second part of the table is the exact formula used to rank the relations for each method.

To perform link prediction, we utilize mentioned ranking formulas derived from the structural properties of the network, such as node degree and neighbor count, as presented in Table 2. The notation Γ(x) represents the set of neighbors of node *x* in the associated network. The cardinality of Γ(*x*), denoted by |Γ(*x*)|, counts the number of neighbors of *x*.

In a simple network, |Γ( x ) ∩ Γ( y )| represents the common neighbors of nodes x and y . In the case of a bipartite graph, our focus is on predicting the relationships between the two parts of the graph. Therefore, our primary objective in this research is not to identify the relationships solely between probiotics or between diseases. Therefore, to discover additional relationships, we will use the notation Γ(Γ( x )) to denote the set of neighbors of neighbors of node x . Similarly, the shared neighbors between node x from one part and the neighbors of neighbors of node y from the other part can be counted using |Γ( x ) ∩ Γ(Γ( y ))|. This measure defines the similarity between nodes x and y in a bipartite network, where x and y belong to different network parts and do not share any direct common neighbors.

Eventually, the best predictor among the link prediction methods based on the formulas listed in Table 2 will be the one with superior results. These unsupervised link prediction algorithms are commonly evaluated using two well-known performance metrics: AUC (Area Under the Curve) and either precision or accuracy AUC and precision^82^. AUC reports the degree of separation between the results and random outcomes, producing a number between 0.5 and 1, with larger values indicating better results. We will use this metric to compare the rank of the selected formulas from Table 2 for two non-existent random edges: one from a test set edge intentionally removed from the network for evaluating the prediction accuracy, and the other from real non-existent edges in the network. The rank of the intentionally removed link that belongs to the network should be greater than the actual non-existent relation randomly removed. So, the greater the difference in rank between the test edge and the randomly chosen non-existent edge, the better the prediction accuracy.

Also, to ensure all available edges are tested, we apply a ten-fold cross-validation mechanism. We randomly select ten disjoint sets of edges to test in ten rounds, removing 10 percent of the current edges in each round. We calculate the area under the curve (AUC) using Eq. (1) and compare it with the rank of randomly selected non-existent edges to identify and report the best-performing link prediction formula. AUC is counted if, out of n randomly chosen pairs of edges, the intentionally removed link for the test has a higher score than the rank of the randomly chosen non-existent link in n’ cases. If both ranks are equal, we apply 0.5n” accordingly in the formula, counting the half of the case and adding it to n’. AUC values range from 0.5 to 1, with higher AUC values indicating better performance of the tested link prediction score function.

1 $$\text{AUC}=\frac{{n}^{\prime}+0.5n"}{n}$$

Similarly, we will report the precision criterion using Eq. (2), which represents the ratio of correct predictions to the total number of changes.

2 $$\text{Precision}=\frac{Correct \; predictions}{All \; Predictions}$$

## Data and methods

Our study focused on human probiotics and associated diseases. We gathered research data from the Probio database (https://bidd.group/probio/homepage.htm), which collects probiotics from various sources, including research, market, and clinical trials. The research probiotics included in our study have reportedly demonstrated beneficial functions in in-vitro, in-vivo, or other laboratory studies. To justify our selection of related diseases, we utilized the ICD-10 (International Classification of Diseases-10th Revision) disease categorization (https://icd.who.int/browse10/2010/en#/), an international statistical classification system for identifying diseases.

**Figure 3:**
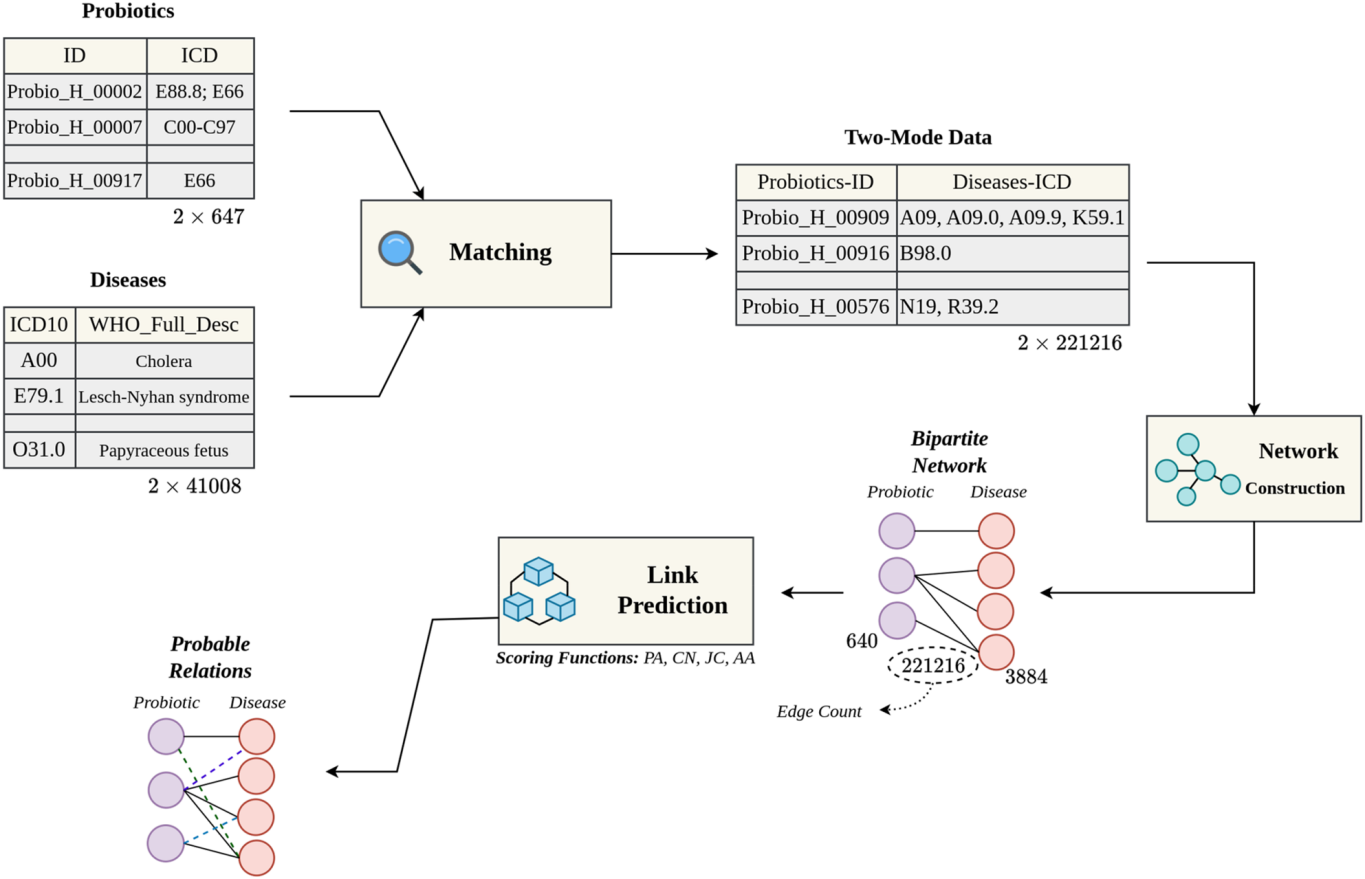
Research workflow for predicting promising probiotic-disease relations.

A brief overview of the research workflow is as follows, Fig. 3. Data was obtained from source website (Probio database) and diseases were assigned to each probiotic based on its ICD code. After constructing a two-mode table of probiotics and their associated diseases, a bipartite network with 221,216 relations was created for link prediction computation, and the best predictor of relations was determined using the results of AA, JC, PA, and AA ranking formulas for that network based on AUC and precision metrics. Therefore, at the final stage of the research process, we have the best ranking list of the most probable probiotic-disease predictions.

Finally, it is also valuable to check the computationally evaluated methods with practical, real-world results. We compare the results of prediction with carefully searching google scholar and science direct recent years data to find the evidence confirming our findings.

## Results

We obtained primary statistics from web scraping and presented them in Table 3. This table includes the number of probiotics and diseases available in the network and the average degree, representing the average number of connections per probiotic or disease. This means that each disease could be impacted by an average of 57 different probiotics. Additionally, we calculated the total number of connections (network relations) between probiotics and diseases and the fraction of available connections to the total number of possible links (network density).

**Table 3:** General properties of the constructed network for probiotic-disease from probiodb and ICD-10.

Dataset property	Number	Average degree
Probiotics	640	346
Diseases	3884	57

Network relations	Network density	
221,216	0.08899	

**Figure 4:**
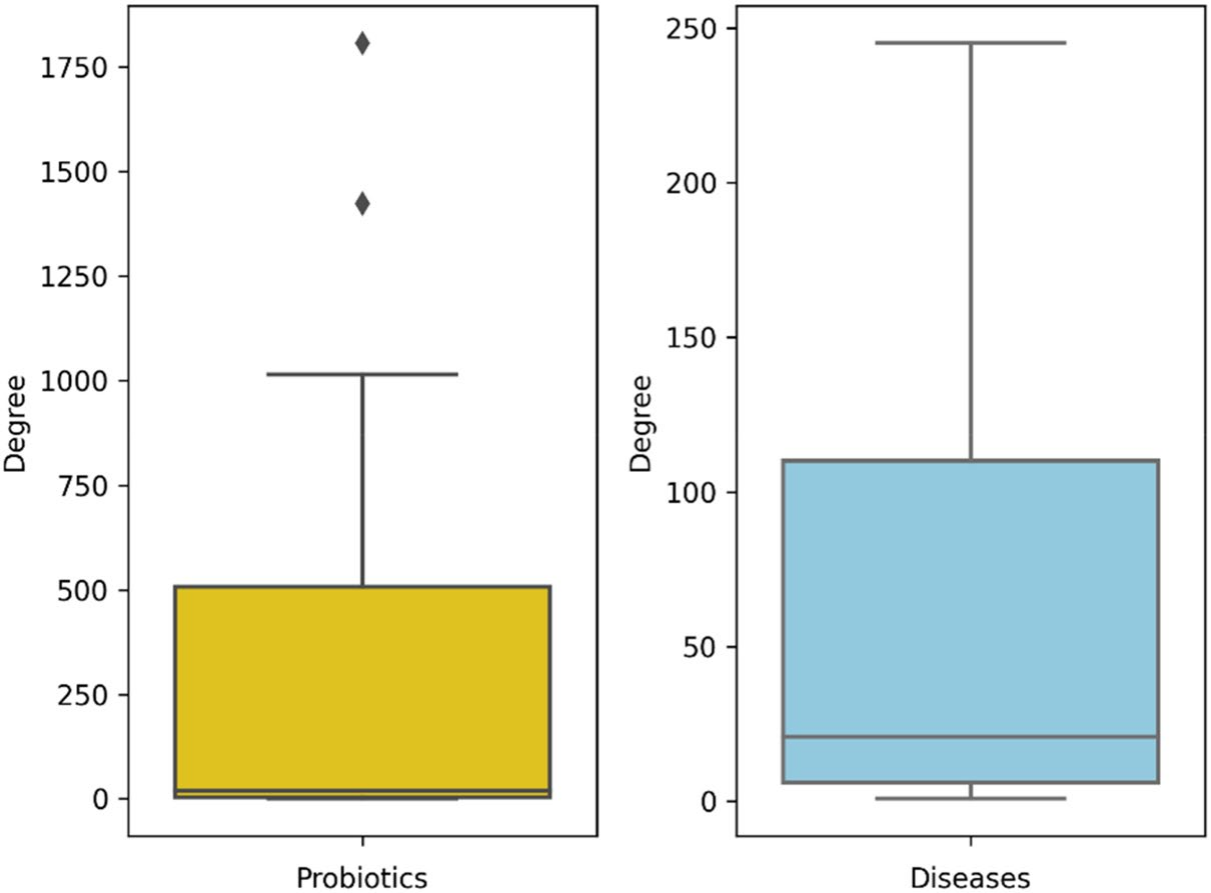
Distribution of the relation numbers and degree, of the probiotics and diseases in the extracted network.

Table 3’s basic statistics indicate that the average number of connected diseases for each probiotic is much more than the typical number of probiotics related to each disease. For more clarification, Fig. 4 presents the boxplots displaying the distribution of the number of relations for probiotics and diseases, revealing that most entities have low degrees of connectivity. Besides, the total network is difficult to depict meaningfully due to the large number of populated nodes and edges.

**Figure 5:**
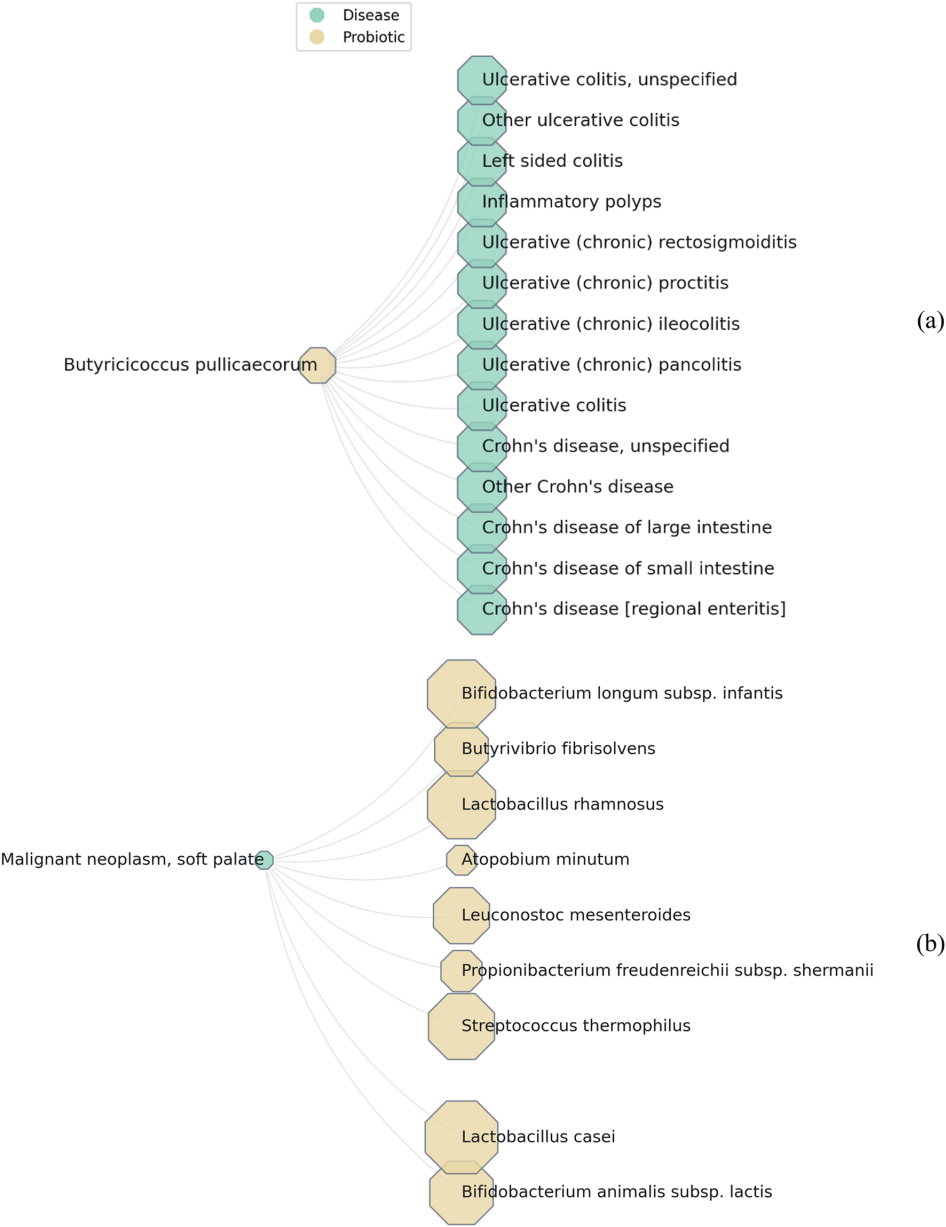
Sample probiotic-disease and disease-probiotic relation visualization in the bipartite network, 5a depicts the *Butyricicoccus pullicaecorum* and its associated diseases, and 5b shows the malignant neoplasm soft palate and its related probiotics.

While showcasing just two basic visualizations, Fig. 5 illustrate the exciting potential of computational methods to unveil meaningful connections between probiotics and diseases. To ensure clarity these examples intentionally feature simplified relationships: Fig. 5a for Probiotic *Butyricicoccus pullicaecorum* and its connected diseases, and Fig. 5b for Malignant Tumors of the Palate and its linked probiotics. The size of each octagon reflects the "importance" of that entity, considering its total connections within the network (other diseases or probiotics).

Based on the Probio database, for Fig. 5a, the probiotic *Butyricicoccus pullicaecorum* shows potential effects on at least 14 diseases linked to inflammatory bowel diseases (IBDs) like ulcerative colitis and Crohn’s disease. Besides, some studies that confirm it may be particularly beneficial for IBD patients^84^. However, it is important to remember that other probiotics might also influence these diseases, which has not been shown in the figure. In the same way, when looking at a specific disease and its network of related probiotics, we can see that different probiotics have varying effects. For example, malignant palate tumors may be influenced by diverse probiotics, including *Lactobacillus casei*, which has a broad range of impacts on other diseases. While, other effective probiotics on this disease, like *Atopobium minutum*, might have a narrower scope of influence on others.

Moreover, based on Table 3, with 640 probiotic strains and 3884 diseases examined, the maximum possible connections between them are 2,485,760, significantly more than the actual 221,216 relations observed. As a result, the network density (the number of existing relations divided by the total number of possible connections) is low. The node neighborhood-based link prediction algorithms utilized in this study rely on network density or the number of connections, structural information, available in the network. Therefore, higher network density leads to more accurate predictions.

Upon examining the constructed network, Table 4 indicates the probiotics and diseases with the highest and lowest connections. This table reveals a considerable pattern: some probiotics, like *Butyrivibrio fibrisolvens*, *Lactobacillus acidophilus*, and *Lactobacillus jensenii*, have significantly more connections to various diseases compared to the average of 346 interactions per probiotic. These “powerhouse” probiotics are relatively few. Interestingly, the diseases that probiotics impact most frequently—Functional diarrhea, Irritable bowel syndrome, and Crohn’s disease—are also the conditions for which probiotics are most commonly prescribed^85^. This demonstrates potential specialization among certain probiotics and focuses on treating particularly prevalent digestive disorders.

**Table 4:** List of probiotics and diseases with the most and least connections to diseases.

Nodes (probiotics and diseases) with the highest degrees (relations)	
1: *Butyrivibrio fibrisolvens*—> 1806	1: Functional diarrhea—> 245
2: *Lactobacillus acidophilus*—> 1423	2: Irritable bowel syndrome with diarrhea—> 196
3*: Lactobacillus jensenii*—> 1423	3: Irritable bowel syndrome without diarrhea—> 196
4: *Lactobacillus gasseri*—> 1014	4: Irritable bowel syndrome—> 196
5: *Bifidobacterium animalis* subsp*. lactis*—> 1014	5: Crohn’s disease, unspecified—> 193
6*: Bifidobacterium breve*—> 1014	6: Crohn’s disease of small intestine—> 193
7: *Bifidobacterium longum* subsp*. infantis*—> 1014	7: Other Crohn’s disease—> 193
8: *Lactobacillus casei*—> 1014	8: Crohn’s disease of large intestine—> 193
9: *Lactobacillus rhamnosus*—> 1014	9: Crohn’s disease [regional enteritis]—> 193
10: *Lactobacillus plantarum*—> 1014	10: Ulcerative colitis, unspecified—> 191
Nodes (probiotics and diseases) with the lowest degrees (relations)	
1: *Lactobacillus reuteri*—> 1	1: Mental and behavioral disorders due to the use of cannabinoids, harmful use—> 1
2: *Streptococcus salivarius*—> 1	2: Anankastic personality disorder—> 1
3: *Bifidobacterium animalis* subsp*. lactis*—> 1	3: Rheumatoid arthritis with involvement of other organs and systems, multiple sites—> 1
4: *Bifidobacterium longum*—> 1	4: Other mental retardation, significant impairment of behavior requiring attention or treatment—> 1
5: *Lactobacillus casei*—> 1	5: Other disorders of pigmentation—> 1
6: *Lactobacillus plantarum*—> 1	6: Transient tic disorder—> 1
7: *Lactobacillus gasseri*—> 1	7: Conduct disorders—> 1
8: *Bifidobacterium longum*—> 1	8: Mental and behavioral disorders due to the use of hallucinogens, harmful use—> 1
*9: Bifidobacterium bifidum*—> 1	9: Anxiety disorder, unspecified—> 1
10: *Propionibacterium thoenii*—> 1	10: Intentional production or feigning of symptoms or disabilities, either physical or psychological [factitious disorder]—> 1

Conversely, the nodes with the lowest degree of relations highlight probiotic strains demonstrating fewer discernible effects on diseases. This observation underscores the need for additional investigation and implies the existence of less-established connections to other diseases. For instance, a review article titled “The role of potential probiotic strains *Lactobacillus reuteri* in various intestinal diseases: New roles for an old player” discusses the role of *Lactobacillus reuteri* and also mentions other probiotics including *Bifidobacterium* spp., *Propionibacterium* spp., and *Streptococcus* spp.^86^.

**Figure 6:**
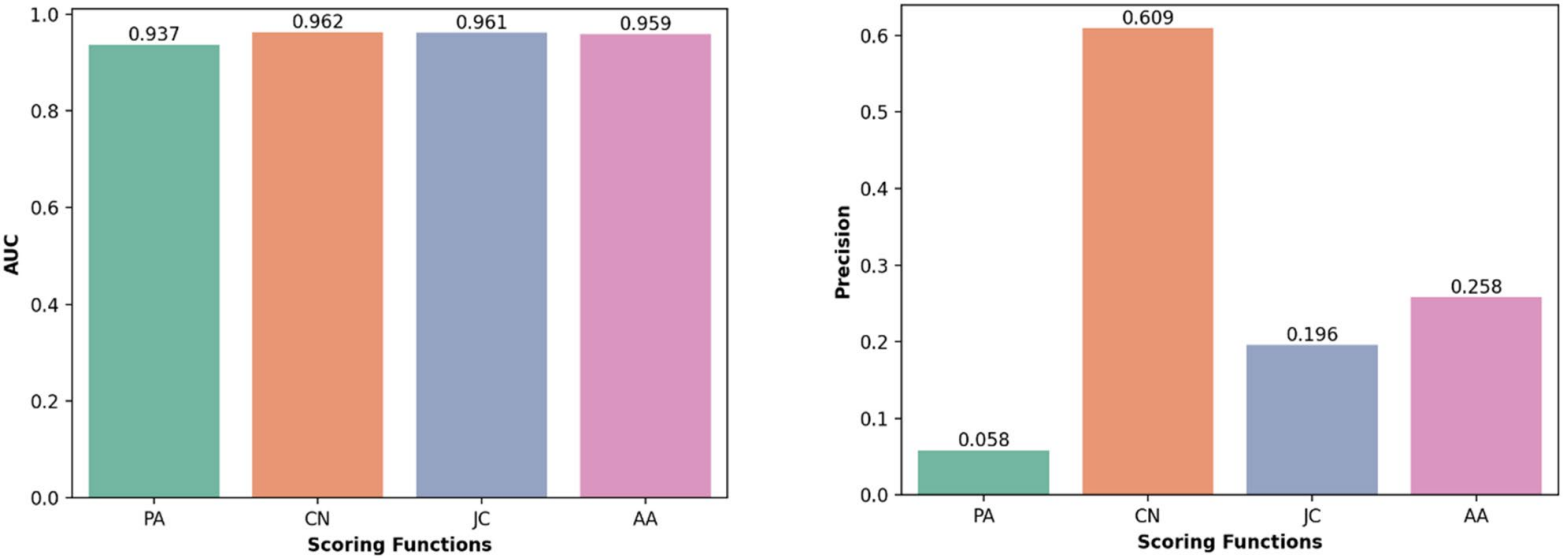
Computational evaluation of link prediction methods in finding new probable relations between probiotics and diseases.

According to precision and AUC metrics, CN is the most effective predictor, with a minimum accuracy of 60% in identifying potential relationships (Fig. 6). This suggests that, based on our evaluation, over 60% of computational results using the CN ranking formula are likely to be accurate. To validate these findings, we suggest exploring the top-ranked relationships identified by CN and searching for recent research that supports these predictions. We conducted a confirmation search on Google Scholar and Science Direct databases for the top 20 CN-ranked predictions and included recently published papers that validate these relationships (Table 5).

**Table 5:** Most promising predictions and their confirmation in recent research.

Probiotic name	Disease name	Google Scholar	Science Direct
*Lactobacillus jensenii*	Functional diarrhea	–	^114^
*Lactobacillus acidophilus*	Acute vaginitis	^115^ ^116^	
*Lactobacillus jensenii*	Ulcerative (chronic) pancolitis	–	–
*Lactobacillus jensenii*	Inflammatory polyps	–	–
*Lactobacillus jensenii*	Other Crohn’s disease	–	–
*Lactobacillus jensenii*	Ulcerative (chronic) rectosigmoiditis	–	–
*Lactobacillus jensenii*	Ulcerative colitis, unspecified	–	–
*Lactobacillus jensenii*	Left-sided colitis	–	–
*Lactobacillus jensenii*	Ulcerative colitis	^102^	–
*Lactobacillus jensenii*	Ulcerative (chronic) proctitis	–	–
*Lactobacillus jensenii*	Ulcerative (chronic) ileocolitis	–	–
*Lactobacillus jensenii*	Crohn’s disease of small intestine	–	–
*Lactobacillus jensenii*	Crohn’s disease of large intestine	–	–
*Lactobacillus jensenii*	Crohn’s disease [regional enteritis]	–	–
*Lactobacillus jensenii*	Crohn’s disease, unspecified	–	–
*Lactobacillus jensenii*	Other ulcerative colitis	–	–
*Lactobacillus acidophilus*	Vaginitis, vulvitis, and vulvovaginitis in infectious and parasitic diseases classified elsewhere	^117,118^	–
*Lactobacillus acidophilus*	Urinary tract infection, site not specified	–	^119^
*Lactobacillus jensenii*	Megacolon, not elsewhere classified	–	–
*Lactobacillus jensenii*	Other functional intestinal disorders	–	-–

## Discussion

This paper leverages well established link prediction methods used in bioinformatics for several reasons. Firstly, applying these well-understood methods to a new domain demonstrates their initial promise for this application. Successful results with basic formulas pave the way for future research on refinements and potential extensions. Secondly, this study focuses on link prediction within a bipartite graph structure. A key contribution lies in adapting unsupervised algorithms to this network type, ensuring a balanced view incorporating predictions from both the “probiotics” and “diseases” sides. Lastly, these established formulas offer appealing advantages^81^: they are computationally efficient, requiring acceptable time and computational resources, and are readily understandable by non-computer specialists within the research field.

In recent years, computational-assisted drug discovery (CADD) has emerged as a powerful approach to expedite the identification and optimization of novel therapeutic compounds. CADD empowers researchers to accurately predict the interactions between small molecules and biological targets by utilizing computational algorithms, machine learning methods, and molecular modeling techniques^87,88^. This predictive modeling not only accelerates the drug discovery process but also enhances the likelihood of identifying candidate compounds with favorable pharmacokinetic properties and therapeutic efficacy^89^.

One potential deficiency in the current research is the lack of incorporating computational modeling approaches to study the regulatory mechanisms and identify potential therapeutic targets in diseases associated with the probiotic-disease network. Ordinary differential equation (ODE)-based theoretical modeling studies on gene/protein signaling networks have proven valuable in understanding regulatory mechanisms and identifying potential therapeutic targets in various diseases^90–92^. These computational modeling approaches could be integrated with the current link prediction methods to gain insights into the underlying regulatory mechanisms and potential therapeutic targets influenced by probiotics in different diseases.

By incorporating ODE-based modeling techniques, researchers can investigate how probiotics modulate gene/protein signaling networks, leading to changes in cellular processes and disease outcomes. These models can simulate the dynamics of gene/protein interactions, identify key regulatory nodes, and predict potential therapeutic targets. Combining such computational modeling approaches with the link prediction methods presented in this study could provide a more comprehensive understanding of the probiotic-disease network, paving the way for developing more effective probiotic-based therapeutic interventions.

In the context of probiotics research, parallels can be drawn between computational-assisted drug discovery and the prediction of probiotic efficacy. Just as CADD facilitates the identification of small molecules with desired pharmacological properties, computational methods can aid in the selection of probiotic strains with optimal health benefits^93^. By analyzing microbial genomes, host-microbiota interactions, and clinical outcomes, researchers can develop predictive models to guide probiotic discovery and development.

Furthermore, the integration of CADD principles into probiotics research holds promise for expanding our understanding of probiotic mechanisms of action and optimizing therapeutic interventions. By applying computational algorithms to analyze microbiome data, researchers can uncover novel probiotic-host interactions and identify key microbial signatures associated with health outcomes. Consequently, there are review researches that highlight the importance of computational approaches in understanding the role of probiotics and microbiota in health and disease, which could potentially inform future drug discovery efforts. Jin et al. ^94^ suggests that multi-omics application is useful in selecting probiotics and understanding their functions on the host microbiome. Also, Niazi and Mariam ^95^ elaborates on the computational works conducted on the microbe–disease and microbe–drug topics. It discusses the computational model approaches used for predicting associations and provides comprehensive information on the related databases.

One of the frequent probiotic strains repeated in the top probable results is *Lactobacillus jensenii*, Table 5. *L. jensenii* is commonly identified in both symptomatic and asymptomatic female urinary microbiota. *Lactobacilli* are known to be dominant members of the healthy female urogenital microbiota^96^. Despite its widespread use, there is currently little scientific evidence to support the efficacy of *L. jensenii* for particular purposes^97^. For example, research has demonstrated that the depletion of vaginal *lactobacilli* is associated with these conditions, including bacterial vaginosis^98^, and trichomoniasis^99,100^. *Lactobacilli* can promote a healthy vaginal environment by producing lactic acid, which helps maintain the normal pH range of 3.8 to 4.5 and prevents bacterial adherence to vaginal epithelial cells^101^.

*L. jensenii* TL2937 is an immunobiotic strain capable of interacting with the immune system^102^. TL2937 has been shown in vitro to suppress nuclear factor κB (NF-κB), which is implicated in the development and progression of various cancers in humans^103,104^. Additionally, TL2937 inhibits mitogen-activated protein kinase (MAPK) signaling pathways, which are involved in the pathogenesis of human disorders, such as cancer and neurodegenerative diseases^105,106^.

Moreover, TL2937 regulates the expression levels of inflammatory cytokines and chemokines upon Toll-like receptor (TLR)-4 activation^107^. It also mediates the induction of negative regulators of TLRs and mitigates intestinal inflammatory damage^106^. These protective effects against intestinal inflammation have also been demonstrated in pigs^102^. It is noteworthy that chronic inflammation is associated with malignancy, and numerous cancers have been linked to chronic inflammation^108–111^. Furthermore, chronic colonic inflammation caused by UC or CD is widely known to increase the risk of colon cancer^112,113^. Given the above explanations and predictions, it is reasonable to infer that *L. jensenii* could be a therapeutic agent for gastrointestinal diseases such as CD and UC. Nonetheless, additional preclinical and clinical studies are required to confirm this hypothesis.

One study approved that *Lactobacillus acidophilus*, predicted as the second row of Table 5, has the potential to use for bacterial vaginosis treatment as it can restore a normal vaginal environment^120^. Moreover, Murina et al. (2011) found that *L. acidophilus* can effectively prevent or reduce vaginal infections, especially recurrent vaginal candidiasis, by maintaining normal vaginal flora. Clinical trials using vaginal tablets containing *L. acidophilus* LA02 in combination with *lactobacillus fermentum* LF10 have shown that they facilitate the development and maintenance of a biofilm that reduces the persistence of Candida infections^121^. However, the role of *L. acidophilus* in trichomoniasis remains unclear. In an animal model, the duration of infection was found to be longer *in L. acidophilus*-treated mice infected with Trichomonas vaginalis compared to the control group^121^. Therefore, further research is necessary to determine the efficacy of *L. acidophilus* in treating trichomonas vaginalis, as per our prediction.

Urinary tract infections (UTIs) are a common health problem in developed countries, affecting 100–180 million individuals annually^122,123^. *Escherichia coli* is the primary cause of uncomplicated UTIs^124^. Unfortunately, antibiotics are frequently prescribed excessively and inappropriately by physicians. This misuse of antibiotics, including overprescription, as well as the administration of ineffective agents, doses, and durations, contributes to the global development of antimicrobial resistance^125,126^. Probiotics, particularly *Lactobacillus*, have shown potential for treating UTIs as part of an alternative or multi-drug therapy due to their antibacterial properties^127^. These effects are mainly attributed to the organic acids produced, pathogen adhesion reduction, and bactericidal properties of *Lactobacilli* bacteria^128–130^. Thus, we predict that *L. acidophilus*, a member of the *Lactobacillus* group, has the potential for UTI treatment.

Computational methods are pivotal in identifying potential therapeutic targets and biomarkers for precision medicine, emphasizing the importance of computational biology’s integration and advancement^131^. These methods are particularly significant in understanding the functional roles of non-coding RNAs in disease biology^132^ and providing insights into genetic markers and ncRNAs associated with the probiotic-disease network. The prediction of miRNA-IncRNA interactions is a key aspect, shedding light on the regulatory mechanisms underlying probiotic-disease relationships. Computational prediction models, such as those developed by Wang et al.^133^, have been instrumental in identifying potential therapeutic targets and biomarkers for various diseases. This model proposes a method named GCNCRF, which predicts interactions between human lncRNA and miRNA accurately. Similarly, literature^134^ presents a model called NDALMA for predicting interactions between long non-coding RNAs (lncRNAs) and microRNAs (miRNAs), which are known to regulate therapeutic targets and diagnostic biomarkers in various human diseases. In the context of probiotic therapeutic effect prediction, this model could potentially be applied to predict the interactions between lncRNAs and miRNAs influenced by probiotics.

## Conclusion and future works

Probiotics are widely recognized for their potential to confer health benefits on humans. The efficacy of probiotics, however, varies depending on the particular strain employed. Computational prediction can provide valuable insights for researchers seeking to identify the most promising probiotic strains targeting specific diseases. This, in turn, can facilitate the discovery of novel, effective alternative treatments for various conditions.

Probiotic-disease predictions suggest that *L. jensenii* may confer prophylactic and therapeutic benefits for gastrointestinal diseases, while *L. acidophilus* may have potential activity against urologic and female genital illnesses. These findings highlight the need for well-designed studies on animals and humans to investigate the effects of these probiotic strains. However, Further studies should aim to predict the effects of probiotics on immune-related gene expression in the context of oxidative stress, given the role of oxidative stress in chronic inflammation. Chronic inflammation, in turn, is implicated in the pathogenesis of numerous diseases, such as Alzheimer’s, asthma, cancer, heart disease, rheumatoid arthritis, and type 2 diabetes.

Several successful applications of link prediction to medical and biological networks have been reported^8,83,135^. However, the scarcity of confirmatory evidence for predicted results may be attributable to the nascent field stage. Because, based on our investigations, experimental confirmation examinations are limited in the literature. An important implication of our study is that each unconfirmed relationship between probiotics and diseases listed in Table 5 represents a promising candidate for in vitro or in vivo testing.

From a computational standpoint, there exist several powerful ranking formulas for link prediction, including path-based, stochastic, and supervised machine learning-based methods. A potential avenue for future research in this area is to leverage stronger link prediction techniques to improve the accuracy of results. However, applying more sophisticated methods may entail increased computation time and complexity, particularly when dealing with big data. Moreover, integrating domain knowledge and node attributes into supervised machine learning-based link prediction algorithms may enhance predictive performance.

Besides, the advancement of interaction prediction research in various fields of computational biology, especially in probiotic-disease relation prediction, holds significant potential for understanding genetic markers and ncRNAs related to the probiotic-disease network. While current research covers a wide range of computational methods for predicting protein–protein interactions and ncRNA interactions, they do not directly address the specific context of probiotic-disease relation prediction. However, the general principles and methods discussed can be applied to studying probiotic-disease relations. For instance, using computational methods for predicting lncRNA-miRNA interactions^134,136^ and the role of ncRNA regulatory mechanisms in diseases^137^ can be valuable in understanding the impact of probiotics on the host’s gene expression and disease outcomes. Additionally, integrating multi-omics data, including genomics, transcriptomics, and proteomics, can provide insights into the interactions between probiotics, the host, and the disease state^94^. Machine learning approaches to understand mechanistic microbiome-host interactions^138^ and the prediction of lncRNA-miRNA interactions^139^ can be relevant to the study of probiotic-disease relations. Overall, the general principles and methods discussed in these papers can be leveraged to gain valuable insights into genetic markers and ncRNAs related to the probiotic-disease network.

Additionally, computational concepts that consider the relationships between two sets of entities as a bipartite network, which hasn’t been applied to the probiotic-disease association, could potentially enhance the algorithms and methods used in current research, leading to improved results^140^. Even if the problem isn’t modeled as a bipartite network, transforming the probiotic-disease bipartite network into two homogeneous networks could result in two networks that can leverage advanced computational methods similar to those used in these studies^141,142^.

Finally, some practical deficiencies in the current research may include limitation of the data sources used for constructing the probiotic-disease network, lack of rigorous lab testing validation of the predicted results, lack of accounting the factors like dosage, delivery method, host health status, etc. than can impact probiotic efficacy, and only considering the relations between probiotics and diseases, not probiotic-probiotic and disease-disease links. These may be covered in future directions as follows:

- Incorporate multiple probiotic and disease databases for a more comprehensive bipartite network.
- Perform lab validation through in vitro and animal studies to test top predicted links.
- Consider different strains, dosages, delivery mechanisms, and host factors in the analysis.
- Expand the network to include probiotic-probiotic and disease-disease connections.
- Integrate other biological data types like genetic markers, gene expression, metabolites, etc.
- Build visualization tools to explore the probiotic-disease network interactively.
- Collaborate with microbiologists to prioritize the most promising predictions for further research.

## Data Availability

All data generated or analyzed during this study are included in this published article.

## Abbreviations

ICD-10: International Classification of Diseases-Tenth Revision
AUC: Area under the curve
WHO: World Health Organization
IBS: Irritable bowel syndrome
IBD: Inflammatory bowel disease
CD: Crohn’s disease
UC: Ulcerative colitis
AR: Allergic rhinitis
VAP: Ventilator-associated pneumonia
miRNA: MicroRNAs
CN: Common neighbors
JC: Jaccard coefficient
AA: Adamic/Adar
PA: Preferential attachment
NF-κB: Nuclear factor κB
MAPK: Mitogen-activated protein kinase
TLR: Toll-like receptor
UTIs: Urinary tract infections
